# “I Felt Like a Burden”: An Exploration Into the Experience of Romantic Relationships for People With ADHD

**DOI:** 10.1111/jmft.70097

**Published:** 2025-11-28

**Authors:** Maidy O'Brien, Christina Kini‐Seery, Caitlin Kelly, Ken Kilbride, Margo Wrigley, Finiki Nearchou, Jessica Bramham

**Affiliations:** ^1^ UCD School of Psychology Belfield Dublin Ireland; ^2^ ADHD Ireland Carmichael Centre for Non‐Profits Dublin Ireland; ^3^ National Clinical Programme for ADHD in Adults, Health Service Executive Dr Steeven's Hospital Dublin Ireland

**Keywords:** ADHD, neurodivergence, qualitative research, rejection sensitivity dysphoria, romantic relationships, thematic analysis

## Abstract

Romantic relationships can have additional complexities for individuals with Attention‐Deficit/Hyperactivity Disorder (ADHD). The present study employed Reflexive Thematic Analysis (RTA) to explore what challenges adults with ADHD have experienced in their romantic relationships. Adults with ADHD (*N* = 355) answered an open‐ended survey question asking if and how their ADHD had negatively impacted their romantic relationships. RTA resulted in four overarching themes and seven subthemes. *Too Much and Never Enough: The Emotional Rollercoaster of Rejection Sensitivity* and *The Struggle for Stability: ADHD's Battle Between Passion and Distraction*, encapsulated the relational challenges individuals with ADHD face associated with their ADHD traits. Between *Partner and Caregiver: The Emotional and Practical Strain in ADHD Relationships* highlighted the sometimes‐unbalanced dynamic in relationships. *From Chaos to Clarity: The Role of Self‐Understanding in Love* explored the detriments of lacking self‐insight and the transformative impact of self‐awareness. Findings underscore the complicated interplay between ADHD traits, self‐perception, and romantic relationships.

## 1 Introduction

Attention‐Deficit/Hyperactivity Disorder (ADHD) is a neurotype characterized by traits of attention regulation differences, hyperactivity, and impulsivity (American Psychiatric Association 2024). Stable and fulfilling relationships play a crucial role in the mental health and overall well‐being of individuals. Individuals with ADHD who experience positive and supportive relationships report lower levels of anxiety, depression, and social isolation (Ginapp et al. 2023; Halkett and Hinshaw 2021; Smusz et al. 2024). The presence of strong, understanding, and accommodating social connections can significantly enhance their overall quality of life (Babinski et al. 2019; Eakin et al. 2004; Fuller‐Thomson et al. 2022). There is a significant recognition of ADHD's impact on interpersonal functioning, particularly within intimate relationships (Barkley and Brown 2008; Ginapp et al. 2023; Huynh‐Hohnbaum and Benowitz 2022; Ramsay 2010). Romantic relationships represent a salient context where the relational consequences of ADHD, and indeed, the internalized narratives that stem from them, are enacted and magnified (Wymbs et al. 2021).

Research reflects frequent reports of misunderstood intentions, emotional dysregulation, and relational conflict among those with ADHD within romantic relationships. For example, partners of spouses of individuals with ADHD report significantly lower intimacy and marital satisfaction (Ben‐Naim et al. 2017). Similarly, a study comparing romantic dyads including an individual with ADHD and without to neurotypical dyads found that the ADHD partnerships reported more conflict, challenges in marital adjustment, conflict resolution styles, and reciprocal evaluations (Öncü and Kişlak 2022). Additionally, there appears to be an elevated risk in romantic relationships for individuals with ADHD, with those with ADHD being significantly more likely to either perpetrate or experience both psychological and physical intimate partner violence to moderate degrees, irrespective of gender and age (Merscher et al. 2025). It is worth noting that Merscher et al. (2025) did not control for co‐occurring mental health difficulties, despite noting significantly higher rates in their group of ADHD participants, and as such, vulnerability of intimate partner violence may be related to mental health difficulties rather than the ADHD. It is important to consider the challenges adults with ADHD may face and potential vulnerabilities to risk that should be assessed and explored.

These experiences, especially when compounded by repetition and in the absence of any reprieve through ADHD‐recognition, can significantly shape one's self‐concept (Ramsay 2010). Over time, adults with ADHD can internalize these relational difficulties and attribute them to personal failure and fundamental inadequacy (Quinn and Madhoo 2014). This has pervasive impacts on how they view themselves in the context of current relationships and as prospective partners. In this way, their fractured self‐concept can become both a product of previous relationships and a lens through which future relationships are interpreted and navigated (Ramsay 2010). For example, a qualitative study with young women with ADHD found that participants felt their low self‐esteem and negative self‐image contributed to poorer communication in sexual situations and an increased fear of rejection, leading to sexual victimization (Wallin et al. 2022).

In addition to low self‐esteem and negative self‐image, several other factors may impact individuals with ADHD's experiences of romantic relationships. Recent research has highlighted that similar to autistic adults, adults with ADHD demonstrate efforts to socially camouflage their ADHD and assimilate (van der Putten et al. 2024). Social camouflaging in women with ADHD is associated with reduced life satisfaction and depressive symptoms (Wicherkiewicz and Gambin 2024). In their qualitative research, Ginapp et al. observed that adults with ADHD report feeling the need to mask around neurotypical individuals (Ginapp et al. 2023), which might affect mixed neurotype romantic relationships.

Similarly, ADHD is associated with increased experiences of rejection sensitivity (Müller et al. 2024), although not within diagnostic criteria for ADHD and not an experience limited to individuals with ADHD. Research has not identified the prevalence rate of rejection sensitivity among adults with ADHD. However, qualitative experiences of criticism and rejection have highlighted how efforts to cope with rejection can be unfavorable and to the detriment of intimate relationships for individuals with ADHD (Beaton et al. 2022a). Therefore, there are several factors that can affect how adults with ADHD experience romantic relationships.

Given these notable patterns, it is valuable to explore how individuals with ADHD experience their ADHD's impact on their romantic relationships. This study aims to investigate how adults with ADHD understand, make meaning of, and navigate their romantic relationships. Furthermore, it seeks to offer a novel contribution to the field by foregrounding the Irish context, which remains underrepresented in existing research. It pays particular attention to how relational histories, self‐perception, and internalized beliefs inform their roles and dynamics within romantic partnerships. By situating participant narratives within both neuroaffirmative and relational psychological frameworks, this study contributes to a more nuanced understanding of intimacy, identity, and connection in the lives of individuals with ADHD.

In the present study, a qualitative methodology was adopted to allow for an exploratory and interpretative analysis of individuals with ADHD's relational experiences. This approach facilitates a deeper understanding of the psychological, emotional, and relational complexities that shape romantic relationships for individuals with ADHD, offering insights that may inform clinical interventions, psychoeducation, and relationship support strategies.

## 2 Materials and Methods

### 2.1 Study Design

A qualitative, phenomenological study was conducted using secondary data obtained via an online survey with adults with ADHD in an Irish context. The original study was a mixed‐methods study exploring the psychosocial needs of adults with ADHD in Ireland. The authors were specifically interested in conducting the study in relation to the Irish context due to the developing landscape of services for adults with ADHD in Ireland. In 2021, the Irish public health service, the Health Service Executive, launched a model of care for the National Clinical Programme for ADHD in Adults, which proposed specialist, tertiary services in response to the lack of services available in Ireland (Raaj et al. 2024). Several specialist public clinics have been launched since the model of care was published. Demand outweighs resources, as evidenced by a recent study investigating referrals to services that identified there are significantly more referrals made to services than the number of appointments they are able to offer (Rudden et al. 2025), and there are ongoing efforts to further develop services to meet the needs of adults with ADHD in Ireland. As the clinical context for ADHD continues to shift in Ireland, we aimed to explore experiences of ADHD within intimate relationships, to raise awareness among clinicians, allowing them to incorporate support for intimate relationships within clinical practice.

The study utilized Reflexive Thematic Analysis (RTA) to analyze the data, with an ontological approach of critical realism and awareness of tendencies caused by the structures, powers, and psychological mechanisms that might have influenced participants' responses (Brown et al. 2021; Houston 2001; Pilgrim 2014).

### 2.2 Participants

Participants, all of whom were individuals with ADHD and who were linked with ADHD Ireland, were recruited through a purposive sampling approach. ADHD Ireland is the leading national support and advocacy organization for people with ADHD in Ireland (ADHD Ireland 2025). There were 430 respondents to the study overall, and 335 of these provided responses to the question on romantic relationships to which this study pertains. The majority of participants were women (68%), 25% were men, and 7% identified as nonbinary. The age range of participants was 19–71 years, and the mean age was 37 years. Most participants (59%) reported that they were diagnosed with ADHD, 38% self‐identified as having ADHD at the time of the study, and 3% did not report if they were diagnosed or self‐identified as having ADHD (it is worth noting that having ADHD was an inclusion criterion of the study, and therefore these participants' responses were not excluded). In an effort to be inclusive and in line with ADHD Ireland's principles, participants who self‐identified as having ADHD were permitted to participate in recognition of the barriers to accessing and receiving a diagnosis of ADHD in Ireland, such as long waiting lists and reliance on private services (ADHD Europe 2020), as well as the challenges faced by minority groups in receiving a diagnosis (Abdelnour et al. 2022). The presence of co‐occurring conditions was not measured.

### 2.3 Procedure

Recruitment was conducted via ADHD Ireland's professional networks, special interest groups, social media platforms, including Facebook, Instagram, and the ADHD Ireland website. Targeted emails were also sent to individuals subscribed to ADHD Ireland's mailing list. Participation in the study was entirely voluntary, and participants did not receive any incentive or reward for their involvement.

The study received ethical approval from Human Research Ethics Committee—Humanities of University College Dublin and in adherence to ethical guidelines for participant protection. Informed consent was obtained from all participants, who consented to their data being archived and used for future research purposes. Participants were provided with a study information sheet outlining the research objectives, their rights as participants, and the voluntary nature of their involvement.

Data collection was conducted via Pavlovia Surveys (Open Source Tools 2024), an online platform for digitally hosting surveys and questionnaires. The survey featured a question asking if and how their ADHD had negatively impacted on their romantic relationships. The survey adopted open‐ended questions to capture a rich perspective of the experience of individuals with ADHD within romantic relationships. To ensure confidentiality and participant anonymity, all identifying information was removed from the data set before analysis.

### 2.4 Analysis

The responses were analyzed using RTA (Braun and Clarke 2021). RTA was chosen due to its flexibility in capturing the complexity of participants' experiences and its alignment with the study's interpretative focus. The first stage of analysis involved immersive engagement and deep familiarization with the data. This required repeated reading of the text to reach a thorough understanding of it. The second stage involved a systematic examination of the data set to identify segments relevant to the research questions and to assign code labels to these data. The third stage entailed constructing patterns of meaning across the data set and generating preliminary themes for them. The fourth stage involved reviewing these themes in the context of their respective extracts as well as the overall data set. The fifth stage centered on refining each theme to ensure that they each carried a distinct conceptual focus. Finally, the sixth stage involved constructing an analytic narrative that would answer the research questions posed.

The study primarily employed an inductive analytical approach with a focus on participants' lived experiences of romantic relationships. Semantic coding was the primary method adopted, meaning that the analysis focused on the explicit content of participants' responses rather than inferring deeper, latent meanings. This analytic process aligned with a critical realist ontological perspective (Zhang 2023). Operating within this framework, participants' experiences are thought to reflect an underlying reality that exists independently. However, subjective interpretation impacts how this reality is understood. Themes are developed through this interpretation of the data (Braun and Clarke 2021). Strategies employed to mitigate subjective meaning‐making and ensure that themes were grounded in participant experience within the data are outlined in a subsequent section.

Participants' quotes are presented with their participant ID, age, gender, and ADHD “status” (formally diagnosed or self‐identified) to contextualize their responses.

### 2.5 Quality Assurance, Reflexivity, and Rigor

The research team consisted of academic, clinical, and applied expertise. M.O.B. was a Trainee Clinical Psychologist, C.K.S. is a postdoctoral researcher specializing in the area of adult ADHD, C.K. conducted her master's thesis on the psychosocial needs of adults with ADHD in Ireland (the primary study secondary data was drawn from), K.K. is the CEO of ADHD Ireland, and M.W. is the Clinical Lead for the National Clinical Programme for ADHD in Adults in Ireland. F.N. and J.B. supervised the research. F.N. is an associate professor and specializes in conducting research related to health and resilience. J.B. is an academic clinical neuropsychologist, and has specialized in adult ADHD. The research team brought a wealth of clinical, academic, and lived experience to the research. All research team members provided feedback on the analysis and development of themes.

Before engaging with the data, the first author (M.O.B.) and research supervisors (F.N. and J.B.) held discussions to explicitly acknowledge and document M.O.B.'s expectations, assumptions, and potential biases. Considering the study's focus on the romantic experiences of adults with ADHD, M.O.B. recognized her pre‐existing familiarity with ADHD‐specific challenges from both clinical and academic experience, as a Trainee Clinical Psychologist conducting the present study for her doctoral training. Clinical Psychology training in Ireland consists of clinical placements and a doctoral thesis, with teaching on research methods within the program. She anticipated that themes might reflect emotional regulation differences, relational instability, rejection sensitivity, and the relational conflict that can arise from executive functioning challenges. These discussions ensured that F.N. and J.B. were aware of these assumptions before data analysis began, allowing them to remain attentive to potential interpretative bias when later reviewing codes and themes.

To further enhance analytic rigor, feedback provided by experts with lived experience (individuals with lived experience of ADHD in the context of intimate relationships and, therefore, extensively knowledgeable on the subject) was incorporated. A draft of the thematic analysis was shared with an ADHD‐involved couple (author C.K.S. and her partner with ADHD), who were invited to review the document and provide input on language and relevance of themes.

## 3 Results

**Figure 1:**
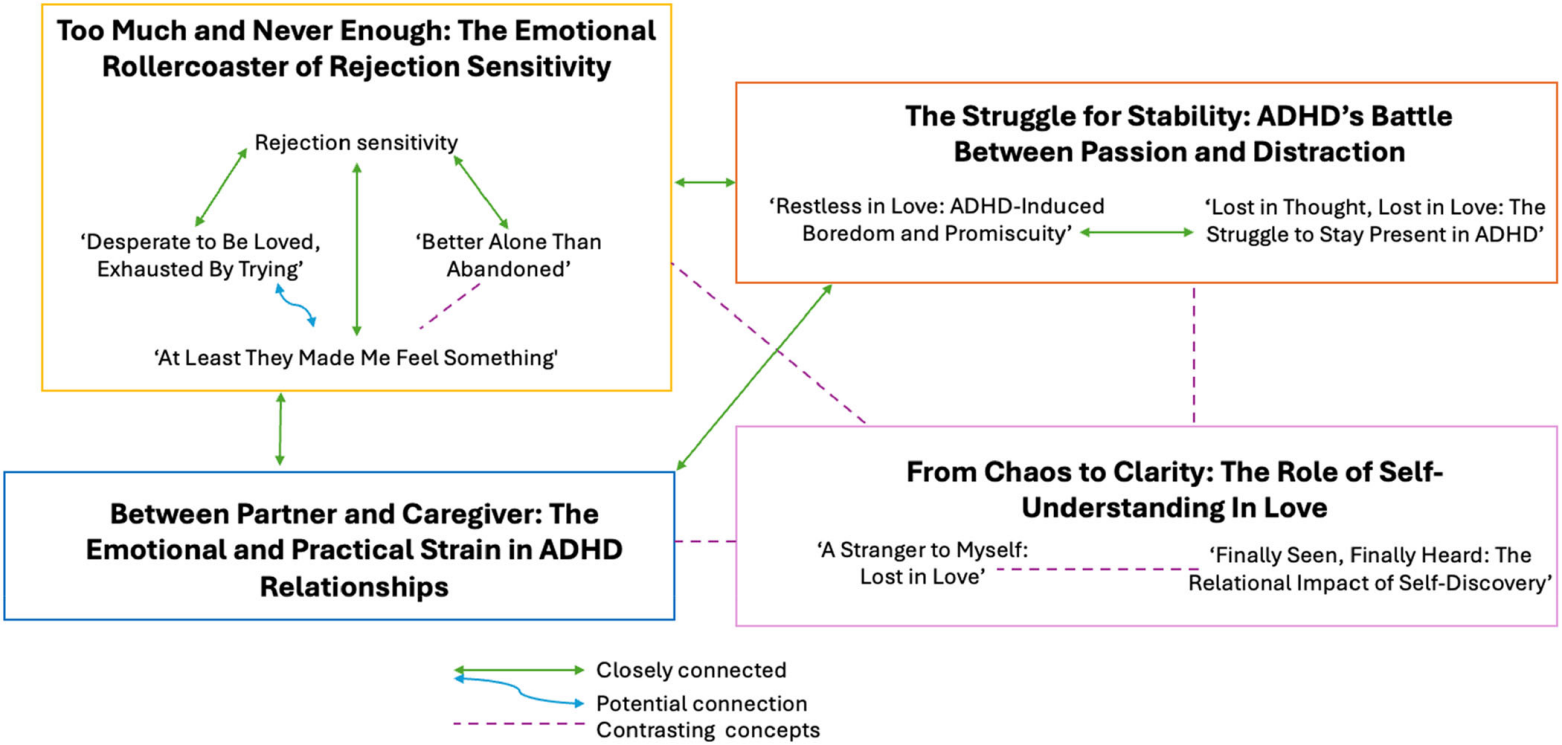
Thematic map of the experience of romantic relationships for adults with ADHD. ADHD, Attention‐Deficit/Hyperactivity Disorder. [Color figure can be viewed at wileyonlinelibrary.com]

Responses (*N* = 335) were analyzed to gain a deeper insight into the experiences of romantic relationships for individuals with ADHD. A thematic structure, comprised of four main themes and seven subthemes (see Figure 1 for visualization of themes, subthemes, and their relationship to one another), was developed as a result (Braun and Clarke 2021).

### 3.1 Too Much and Never Enough: The Emotional Rollercoaster of Rejection Sensitivity

This first theme describes participants' oscillation between overwhelming devotion and withdrawal within romantic relationships.

*Desperate to Be Loved, Exhausted by Trying*. Participants described an excessive prioritization of their partner's needs in an effort to maintain relational security. Their profound need for validation, driven by rejection sensitivity, often led to relational overinvestment, a lack of boundaries, and ultimately, emotional burnout. One participant conveyed the extent of this exhaustive effort, stating, “I would overcompensate by cooking and cleaning a lot and sometimes being ‘OCD’ as a result, but then sloppy a next day. Eventually being punished for being me. It's very painful knowing you're an inconsistent person” (P352, man, 28, formally diagnosed).

Despite their well‐intentioned efforts, participants often found that their behavior was perceived as overwhelming or excessive by their partners. Several participants reflected on this, expressing, for example, “I was too intense for my partners,” (P24, man, 32, formally diagnosed) and “always went in full steam and … they would feel overwhelmed” (P42, woman, 52, self‐identified). These perspectives provide context to the internalization of failure many respondents experienced.

This unrelenting effort to maintain connection while also meeting the demanding needs created by Rejection Sensitivity Dysphoria (RSD) frequently placed strain on respondents' relationships. One participant described the toll this took, sharing, “I put partners through a lot because of my jealousy and need for reassurance” (P413, woman, 54, formally diagnosed). For some, their RSD extended beyond reassurance‐seeking and resulted in fixation and emotional overdependence. The impulsive and overwhelming nature of RSD reactions was described by one participant as follows:

Disproportional sensitivity to rejection or perceived rejection. I used to fly off the handle and not be able to control my reactions, then get really upset and cry and beg for forgiveness.

(P290, woman, 37, formally diagnosed)

Overall, while participants' relational strategies may have provided temporary relief to their RSD, their unsustainable nature frequently led to relational instability, burnout, and ultimately, relationship breakdown.

*Better Alone Than Abandoned*. While the previous subtheme illustrated the overcommitment that fear of rejection often motivated participants to engage in, our second subtheme explores the opposite. Some participants revealed that the fear and pain of rejection were so unbearable for them that they opted to avoid relationships altogether. For instance, one participant described feeling “too afraid to put myself out there for fear of rejection” (P313, woman, 31, self‐identified).

While this participant described a more intense longing for a romantic partner than their peers, other participants expressed how their rejection sensitivity inhibited romantic feelings from developing altogether. One participant reported, “it took me years to feel romantic feelings, but maybe that was a fear of rejection” (P137, woman, 24, formally diagnosed). The safety of romantic avoidance was captured as a dominant thread throughout participant responses, where their perceived inevitability of rejection frequently overshadowed their desire for connection.

However, for the participants who overcame this fear and did enter into romantic relationships, their rejection sensitivity continued to play a pivotal role. Participants' RSD was seen to shape relationship dynamics and limit romantic expression and commitment. For instance, one participant revealed that they “had a string of casual flings, preferring to keep things getting any closer to avoid rejection” (P141, woman, 53, self‐identified). Despite this participant overcoming their RSD enough to pursue relationships, this pre‐emptive distancing strategy still reinforces a cycle of emotional self‐protection at the cost of true connection and intimacy.

This pattern of pulling away from romantic partners was echoed by other participants who shared that they had a tendency to “push them away because I never felt good enough” (P136, woman, 49, self‐identified). For many participants, there was evidence that the avoidance borne from their rejection sensitivity impacted them at every stage of romance.

*At Least They Made Me Feel Something*. Numerous participants expressed the lure of intensity often found within unhealthy relationships. The hallmark of many toxic romantic relationships involves “love‐bombing”—a strategy that consists of an excessive and premature display of affection in an effort to achieve rapid emotional intimacy and control. This flood of validation and affection can be alluring to individuals with ADHD, as it offers them the validation and reassurance they so often crave. However, their deep need for this approval often blinded participants to its coercive and manipulative nature. One participant expressed this lack of recognition by stating,

I was never able to spot ‘red flags’ in a relationship, I would crave the attention & affection from the start and not be able to see if a relationship was a good one.

(P66, woman, 47, formally diagnosed)

Another participant echoed this sentiment, explaining that the initial rush of excitement often delayed assessment of a partner's character. They stated, “I tended to fall head over heels over someone I liked and only really learned who they were when it was too late” (P108, woman, 59, self‐identified). The ease with which participants entered relationships was often closely correlated to their own level of need for acceptance. For some participants, this resulted in maintaining relationships that were unfulfilling.

For others, their unrelenting and compulsive craving for acceptance and approval left them vulnerable to more dangerous relationships. One participant described being “attracted to love bombing and toxic traits. Been in abusive relationships, had to get a protection/safety order” (P2, woman, 37, formally diagnosed). Another reported that their all‐consuming need for validation “led to being in a coercively controlling relationship and some risky behaviour” (P61, woman, 39, formally diagnosed). Another identified this pattern by saying, “I have gravitated towards abusive men” (P252, woman, 33, formally diagnosed). While some participants expressed how this validation‐seeking facilitated the establishment of unhealthy relationships, others described how it also maintained them. For instance, one participant shared, “I felt like a burden to my partner. I was told nobody else would put up with me.” (P351, woman, 32, formally diagnosed). This fear of not having an alternative partner to meet these intense and incessant needs proved enough to sustain destructive and exploitative relationships.

### 3.2 The Struggle for Stability: ADHD's Battle Between Passion and Distraction

The second theme encapsulates the ongoing battle described by many participants between the enticement of novelty and the threat it poses to relational stability.

*Restless in Love: ADHD‐Induced Boredom and Promiscuity*. A common theme among participants' narratives was the intense excitement experienced at the beginning of relationships followed by the inevitable wane of these feelings. One participant summarized this experience by stating, “Often get excited at the beginning, spend loads of time with them, and lose interest rapidly once the honeymoon stage ends” (P16, woman, 24, formally diagnosed).

For some participants, this decline in intensity led to a need to seek novelty and excitement elsewhere. For instance, one participant reported, “Got bored easily and moved on looking for something new” (P105, man, 46, formally diagnosed). Numerous participants expressed their struggle with the transition from newness and novelty to familiarity and routine and how they sometimes managed this by seeking out alternative relationships. For example, “super impulsive. Regularly cheated in past relationships” (P285, woman, 35, self‐identified), and similarly, “always distracted and looking for something new” (P38, man, 44, self‐identified).

Of interest, some participants explicitly referenced how this relational cycle was not indicative of a lack of commitment to their partner. Instead, the accounts speak to the extent to which individuals with ADHD experience impulsivity and sensation‐seeking. For instance, “I was engaged to be married 5 times before I eventually married as I found myself losing interest in partners even though I was committed to the relationship” (P132, man, 58, formally diagnosed). Similarly, another participant shared that they “could get bored or be unfaithful on some stupid impulse and regret that so much” (P359, woman, 55, formally diagnosed). This voice also captures the immediate backlash that individuals with ADHD can face in response to their own unrelenting urges. One participant acknowledged their promiscuity as a dopamine‐chasing exercise, stating “I got bored and found dopamine elsewhere” (P401, woman, 36, self‐identified).

Other participants, however, shared that their pursuit of novelty and impulse for change often eclipsed the fondness and affection they initially felt for their partner. For instance, one participant noted, “I would get very strong emotions and hyperfocus and then I would get fed up or bored and would be inattentive and seem distant” (P249, man, 56, self‐identified). This captures the intensity of both emotional extremes and the all‐or‐nothing nature of some participants' relational experience. This sentiment was conveyed by another participant who shared, “my relationships have ended with me slowly drifting away mentally, my feelings suddenly switching to platonic or even to absolutely not wanting anything to do with them” (P97, woman, 26, formally diagnosed).

*Lost in Thought, Lost in Love: The Struggle to Stay Present in ADHD*. Another dominant thread in participants' accounts was how their inattentiveness was perceived by their partners and how this led to relational complications. This was illustrated by one participant who stated, “forgetfulness and lack of focus creates an impression I am not interested or do not care about partners life and interests” (P396, man, 42, formally diagnosed). This interpretation of ADHD‐related inattentiveness as emotional unavailability was expressed by numerous participants. Another shared, “I forgot plans or things that my ex partner felt were important for something less important on a whim” (P47, woman, 36, formally diagnosed) and “It could seem like I wasn't listening or taking things on board” (P153, man, 46, formally diagnosed). This inattentiveness became a source of relational tension among participants, with one sharing, “I was always told I didn't give enough attention to them” (P199, woman, 50, formally diagnosed).

Moreover, participants explained how their inattentiveness disrupted intimate and emotional connection. For instance, “issues staying focused while being intimate caused partner to query if I was even interested or thinking of someone else” (P104, woman, 36, self‐identified). The response shone a light on the relational strain and emotional neglect partners of participants experienced as a direct result of their ADHD‐related attention difficulties. Participants' inattentiveness also impacted their ability to demonstrate commitment and consistency within their relationships. For example, “I would forget to return calls or answer texts. I couldn't remember some things they told me” (P199, woman, 50, formally diagnosed) and “time‐blindness can make it easy to drift away from people” (P370, nonbinary person, 29, formally diagnosed).

### 3.3 Between Partner and Caregiver: The Emotional and Practical Strain in ADHD Relationships

The third theme captures how the executive functioning challenges and emotional regulation difficulties that characterize ADHD can threaten the balance and mutual reciprocity that romantic relationships rely on.

Some of the demanding aspects of ADHD can result in partner overcompensation, whereby they take on an unequal share of emotional and logistical responsibilities. One participant recognized this disproportion in stating, “it is a very imbalanced relationship and my partner feels as though he has to do everything” (P264, woman, 26, formally diagnosed).

This imbalance can be exacerbated by executive functioning difficulties, which affect the individual with ADHD's ability to manage household tasks and maintain daily responsibilities. One participant explained, “I always found myself stuck in a cycle of leaving chores undone only to feel like an absolute failure and criticised when mentioned” (P104, woman, 36, self‐identified). Another stated, “I'm disorganised, have terrible time management skills” (P346, woman, 47, formally diagnosed), while another referenced their financial struggles, noting “spending issues, low‐level debt” (P354, woman, 36, formally diagnosed). These experiences illustrate how non‐ADHD partners may feel obligated to assume control over household management and decision‐making, reinforcing the imbalance.

The extensive support partners sometimes provide can become overwhelming and, over time, unsustainable. This support often extends beyond practical matters and can involve substantial levels of emotional support for co‐occurring mental health conditions. One participant acknowledged, “I also require a huge amount of emotional support for my anxiety, depression, and general day‐to‐day life. It is exhausting for both of us” (P264, woman, 26, formally diagnosed). Similarly, another participant also referenced their “over‐reliance on partner for emotional labour” (P396, man, 42, formally diagnosed). These accounts perceive an exhaustion experienced by their partner in their efforts to manage both the emotional and practical aspects of the relationship. Previous research has identified an association between depressive symptoms and reduced relationship satisfaction and increased relationship stress (Doyle et al. 2021; Whisman et al. 2021). It may be that the co‐occurring mental health difficulties experienced by some adults with ADHD exacerbate the burden they believe their partner experiences because of their relationship. Over time, this additional labor can evolve into what is experienced as unsustainable levels of caregiving.

Individuals with ADHD reported finding the support provided by the non‐ADHD partner as distressing at times. The perception of being inconsistent, thoughtless, or unreliable can significantly impact their self‐concept. One participant noted, “I can become so engrossed in what I am doing that I will not do something that I said I would do, like make dinner” (P391, woman, 32, self‐identified). Another added, “lack of focus slows domestic tasks” (P396, man, 42, formally diagnosed).

Internalizing these difficulties as a belief that they are somehow fundamentally incapable of being adequate partners perpetuates their feelings of inadequacy, which only exacerbate the imbalance. As a result, some adults with ADHD may develop an increasing dependency on their partner for functional stability. In some instances, what can begin as support can morph into a loss of autonomy for the individual with ADHD as their partner has learned to dictate decision‐making and household management. One participant provided insight into the impact of executive dysfunction on daily life, sharing, “I am constantly misplacing things and asking for help finding them. This could be keys, my glasses, phone, etc.” (P85, woman, 48, self‐identified).

For those who experienced this dynamic deepening, a sense of learned helplessness can emerge, wherein the individual with ADHD no longer relies on their own ability to handle responsibilities independently. One respondent described having, “no trust or confidence in myself to maintain standards required to maintain a successful romantic relationship” (P170, man, 45, formally diagnosed). Another participant described this progression by saying, “partner scaffolding progressed to toxic codependency” (P34, woman, 54, formally diagnosed). This quote illustrates how well‐intentioned support can gradually shift to codependency where the individual with ADHD has not retained independent skill in decision‐making and problem‐solving.

### 3.4 From Chaos to Clarity: The Role of Self‐Understanding in Love

The fourth theme captures the confusion and frustration experienced by participants in response to their own behavior in romantic relationships. It highlights both the distress of not understanding oneself and the transformative power of finally gaining that understanding.

*A Stranger to Myself: Lost in Love*. This subtheme represents the struggle participants encountered to articulate their inner world and behavioral motivations. It conveys the self‐doubt and relational tension that this lack of awareness resulted in, and how they often felt lost and ashamed as a consequence.

Many participants outlined their struggle to make sense of their own actions stating, “I had no idea what I was doing” (P87, man, 53, formally diagnosed) and “I just did not have any great understanding of the struggles I was going through and in particular, rejection sensitivity dysphoria” (P228, woman, 28, formally diagnosed). This experience of disillusion with their own relational performance typically reinforced previously discussed phenomena of self‐doubt, rejection sensitivity, and unworthiness. This was encapsulated by one participant who expressed, “I just accepted that I was a rotten girlfriend and wife” (P115, woman, 55, formally diagnosed). This defeat in the face of relational strain was often compounded by participant's own inability to recognize their ADHD‐specific struggles and express them to their partners. As one participant reported, “I didn't understand, I couldn't and still struggle to explain” (P365, woman, 33, formally diagnosed). This experience was shared by another respondent who stated:

I didn't know how to express my needs and end up in a relationship with someone that could not understand my mood swings or my traits of lack of focus, small mistakes or forgetfulness.

(P272, woman, 27, self‐identified)

This account references the emotional dysregulation and inattention that characterize ADHD. However, this participant, along with many others, did not have the knowledge or awareness to understand or explain their behavior within the framework of neurodiversity.

Participants' lack of understanding of their ADHD‐specific traits and challenges meant that they also lacked ADHD‐specific strategies to manage these difficulties. This lack of guidance was illustrated by one participant who stated, “I didn't know how to stop being late or messy and disorganised or over‐emotional” (P115, woman, 55, formally diagnosed). Another participant shared, “I end up in abusive relationships. I haven't figured out the why” (P11, woman, 43, self‐identified). This account demonstrates how a lack of recognition of ADHD‐involved relational patterns left participants ill‐equipped to break unhealthy cycles. Without awareness of this recurring attraction, individuals with ADHD may mistake unconscious patterns for genuine connection.

Beyond shaping relational choices and behavioral patterns, this lack of self‐awareness also entrenched feelings of disconnection, which left many participants struggling to feel truly seen or understood in their relationships. The sense of feeling unseen, unheard, and misunderstood was expressed by one participant who reported, “I struggled deeply with relationships. I have never felt that another person truly knew me, got me, or saw me for who I am” (P97, woman, 26, formally diagnosed) and by another who described, “20 years of not being heard/understood” (P220, woman, 51, self‐identified). The isolation brought about by this persistent disconnect was illustrated by another participant who shared, “If I'm honest, I have felt lonely most of my life in that nobody understood me, not even my family” (P195, man, 39, formally diagnosed). For many, feeling emotionally out of reach to their partners not only strained intimacy, stability, and commitment but also compounded an internal struggle with self‐identity. One participant described this by saying that they, “felt really unable to ‘unlock’ myself” (P137, woman, 24, formally diagnosed). For more, this unstable self‐concept led to a reliance on romantic relationships. As one respondent expressed, “I couldn't go a month without a boyfriend because my sense of self was non‐existent” (P255, woman, 30, formally diagnosed). Without a clear sense of who they were, relationships transitioned from a source of connection to a necessary anchor for self‐identification.

While some participants described struggling to be themselves, others expressed reproach for doing just that. One participant described this as, “eventually being punished for being me” (P352, man, 28, formally diagnosed). The profound and pervasive impact of ADHD meant that participants sometimes internalized a sense of never being “enough” or that they were somehow fundamentally flawed. This bled heavily into their self‐concept, as illustrated by one participant who shared, “I didn't know that I wasn't a lazy, useless person who deserved to be abused” (P252, woman, 33, formally diagnosed). This internalized sense of inadequacy became a defining feature for how many respondents navigated romantic relationships. This deep‐seated sense of unworthiness was further reinforced by the way participants' behaviors were frequently misinterpreted in their relationships.

*Finally Seen, Finally Heard: The Relational Impact of Self‐Discovery.* This subtheme depicts the powerful shift in perspective brought about by diagnosis or through finding a neurodivergent partner (neurotype that deviates from the assumed norm or expected functioning, such as ADHD, autism, dyslexia, or dyspraxia; Chapman and Botha 2023). The subtheme also covers retrospective understanding and the self‐compassion this generates.

For some participants, later‐life understanding and recognition of ADHD traits alleviated their psychological burden. Participants' narratives demonstrated how informed reflection allowed respondents to retrospectively attribute relationship breakdowns to undiagnosed ADHD rather than personal failure. For example, one respondent noted that they, “lost a relationship because of what I now know as ADHD traits” (P9, woman, 37, formally diagnosed). Another participant stated that “not understanding that I have RSD made everything so much harder” (P79, woman, 47, self‐identified). These accounts illustrate how understanding can mitigate self‐blame which, in turn, can improve relational confidence, and consequently, relational stability.

Participants who disclosed romantic success postdiagnosis or ADHD identification exemplified this shift. One such account was, “I didn't have a serious relationship until after I had a diagnosis and am now married. But it's likely my ADHD affected my dating style” (P165, woman, 33, formally diagnosed). Another respondent conveyed how understanding their ADHD allowed them to unmask their authentic selves. They stated, “being heavily masked, I was not working from my real self so found it impossible to ask or expect my needs to be met by romantic partners” (P356, woman, 48, self‐identified). For another participant, being diagnosed meant they wanted to unmask, which ultimately led to the end of a relationship:

I was in a relationship for 17 years with my ex (undiagnosed autistic) but ended it around the time of my diagnosis and massive burnout. I no longer wanted to mask and pretend all was ok when it wasn't but have also got issues with confrontation and reading my thoughts/emotions correctly.

(P47, woman, 36, formally diagnosed)

For some, relational experiences were transformed through understanding not only their own neurodivergence but by experiencing a partner's. In this way, finding a fellow neurodivergent partner provided an alternative route to self‐acceptance. One respondent described how an ADHD–ADHD relationship offered a dynamic that felt more sustainable, stating, “always had to move on, got bored until I met my wife (who also has ADHD)” (P353, man, 46, formally diagnosed). Another participant reflected on the solace they found in a similarly neurodivergent partner, explaining:

I never managed to get into a romantic relationship up until coming to university, where I still didn't manage to get into relationships until I met someone who was similar to me (my partner probably has ADHD or some sort of neurodiversity) and that's why they tolerated me.

(P335, man, 21, formally diagnosed)

This relief and self‐acceptance were echoed in other accounts that shared:

I'm with someone for 8 months now and he also has ADHD (diagnosed) … He understands the way I think and I'm very happy with him, it's the first time I've felt I don't have to pretend my way through the day.

(P197, woman, 32, self‐identified)

Overall, this subtheme highlights the importance of self‐understanding and re‐contextualizing traits as ADHD, as well as having a romantic partner who understands ADHD. Recognition of the role of ADHD in their challenges can foster self‐compassion and can give adults with ADHD permission to not hold themselves to the same standards as their neurotypical counterparts.

## 4 Discussion

The present study explored the qualitative experience of how ADHD impacts on romantic relationships. Four themes were developed as a result of RTA. The first theme, “Too Much and Never Enough: The Emotional Rollercoaster of Rejection Sensitivity,” captured the emotional intensity and volatility experienced by individuals with ADHD in romantic relationships, particularly in response to RSD. The second theme, “The Struggle for Stability: ADHD's Battle Between Passion and Distraction,” represented the difficulty of attention regulation differences and increased need for novelty and stimulation caused in their relationships. The third theme, “Between Partner and Caregiver: The Emotional and Practical Strain in ADHD Relationships,” illustrates how individuals with ADHD could perceive a disproportionate share of responsibility in their relationships. Finally, the fourth theme, “From Chaos to Clarity: The Role of Self‐Understanding in Love,” reflected individuals with ADHD's experiences of both the absence and presence of self‐awareness and how both distinctly defined their relational experiences. These findings provide valuable insight into ways of supporting individuals with ADHD to thrive in their romantic relationships.

“Too Much and Never Enough: The Emotional Rollercoaster of Rejection Sensitivity” highlighted the significant difficulties of emotion regulation and rejection sensitivity for adults with ADHD in their romantic relationships. The subtheme “Desperate to Be Loved, Exhausted by Trying” reflects the impact of emotional regulation differences experienced by individuals with ADHD. Participants described fearing rejection in their relationships and the urge to overcompensate to reduce the risk of rejection. This ultimately led to burnout or overwhelming their partners. Beaton et al. similarly found that adults with ADHD are intensely fearful of criticism and report that experiences of criticism and rejection had altered their sense of self negatively (Beaton et al. 2022a). Likewise, “Better Alone Than Abandoned” showcased an alternative coping strategy to rejection or criticism, by distancing from and avoiding romantic relationships or engaging in short‐term, casual relationships. Previous research has identified that adults with ADHD are more likely to engage in hypersexual behaviors and sexual risk‐taking and that this is linked to emotion regulation differences, impulsivity, and oppositional symptoms in women with ADHD (Hertz et al. 2022). The present study's findings highlight that an individual with ADHD may be disengaging from romantic relationships as a compensation strategy for the difficult experiences of rejection sensitivity. As such, it is important to cultivate self‐compassion in adults with ADHD, to support them to respond to their rejection sensitivity with self‐kindness and meaningfully engage in romantic relationships if they desire to. The final subtheme “At Least They Made Me Feel Something” suggested that some participants were more at risk of experiencing abusive behaviors in their romantic relationships. This finding is supported by a recent study, which has identified that adults with ADHD are significantly more likely to perpetuate or experience intimate partner violence than neurotypical peers, although the potential confounding variable of co‐occurring mental health difficulties was not accounted for (Merscher et al. 2025). Therefore, it is important that clinicians working with individuals with ADHD are conscious of the risk in romantic relationships.

In “The Struggle for Stability: ADHD's Battle Between Passion and Distraction,” participants reported the challenge of needing novelty for stimulation and how they would feel bored and impulsive once the honeymoon phase had ended. The subtheme, “Lost in Thought, Lost in Love,” reflects some of the relational difficulties linked to attention regulation differences in ADHD, such as differences in active listening, partner understanding, and conflict resolution (Eakin et al. 2004; Öncü and Kişlak 2022). While participants' experiences of impulsivity can be challenging in terms of seeking novel stimulation, Soares et al. have found that ADHD traits are linked to heightened experiences of passionate love (Soares et al. 2021). As such, ADHD traits, such as emotional intensity, can deepen romantic connections and strengthen commitment (Huynh‐Hohnbaum and Benowitz 2022). Given that intimacy mediates the negative impact of ADHD on relationship satisfaction in non‐ADHD partners (Ben‐Naim et al. 2017) and that adults with ADHD experience a heightened intensity of passionate love (Soares et al. 2021), fostering intimacy and excitement in romantic relationships may be helpful for supporting the challenging aspects of impulsivity and attention regulation differences.

“Between Partner and Caregiver: The Emotional and Practical Strain in ADHD Relationships” explored how participants feared their executive functioning differences impacted their relationship and could create an unbalanced dynamic. Participants described internalizing their challenges, like with domestic chores or attention regulation differences, and how that affected their self‐concept in the relationship. Previous research has identified significantly lower levels of self‐compassion and higher rates of negative self‐esteem (Beaton et al. 2022b; Pedersen et al. 2024), reflecting how participants in the current study felt their inadequacies in their relationships impacted how they viewed themselves. Participants reported how their partners could overcompensate for their executive functioning differences, and that this could affect the perceived balance in the relationship. A qualitative study of women without ADHD in romantic relationships with men who have ADHD similarly found that their participants felt they had to take on responsibility for their partner's ADHD, leading to the man with ADHD becoming passive in the relationship (Zeides Taubin and Maeir 2024). Our findings highlight that women and men with ADHD notice this dynamic in their relationships, and the negative consequences of the imbalance inter‐ and intrapersonally. Overall, results suggest the importance of providing supports to adults with ADHD for their executive functioning differences, to address unequal dynamics and thrive in their romantic relationships. Cognitive Behavioral Therapy (CBT) in particular has demonstrated significant effectiveness in supporting adults with ADHD with their executive functioning and mental health (Jensen et al. 2016; Knouse et al. 2017; Young et al. 2020).

The fourth, and final theme, “From Chaos to Clarity: The Role of Self‐Understanding in Love,” reflected individuals with ADHD's experiences of both the absence and presence of self‐awareness, and how both distinctly defined their relational experiences. “A Stranger To Myself: Lost in Love” emphasizes the personal costs of being undiagnosed or not knowing they had ADHD, and how they could not understand their ADHD traits in the context of romantic relationships. Long and Coats conducted a qualitative systematic review of the experience of receiving a diagnosis of ADHD in adulthood. They similarly found that before adults were identified as having ADHD, they experienced a sense of always feeling different from other people, believing there was something wrong with them, and a chronic sense of failure (Long and Coats 2022). They also identified the positive impact of being diagnosed, similar to the subtheme “Finally Seen, Finally Heard: The Relational Impact of Self‐Discovery.” Participants in the present study described how, following their diagnosis, they understood their ADHD traits better, leading to a shift in both how they viewed themselves and navigated their romantic relationships.

The newfound understanding led to some participants realizing the extent of their masking and wanting to unmask. While it is valuable to unmask, as masking ADHD traits can have significant mental health tolls, and is linked to depressive and reduced life satisfaction (Wicherkiewicz and Gambin 2024), one participant identified negative consequences of unmasking and how this led to her separation. Previous research has found that, in a small sample, 58% of non‐ADHD men had ended their relationship with a spouse with ADHD, while only 10% of non‐ADHD women had left their relationship with a husband with ADHD (Minde et al. 2003). Therefore, with this understanding and important desire to unmask, adults with ADHD may require support in navigating their romantic relationships postdiagnosis to thrive. A trial of psychoeducation for individuals with ADHD and their significant others observed a trend toward reductions in burden of care, emotional overinvolvement, and perceived criticism (Hirvikoski et al. 2017). A similar pilot evaluation of group CBT for ADHD integrated with couples therapy observed preliminary improvements in ADHD‐related executive functioning and reductions in relationship negativity (Wymbs and Molina 2015). It would be valuable to further explore the benefits of dyadic psychoeducation on ADHD postdiagnosis to foster a safe environment for individuals with ADHD to unmask and increase understanding in their partners. Several books, both self‐help and aimed toward clinicians, on the topics of ADHD and intimate relationships could support these interventions (Pera 2016, 2023). Additionally, within “Finally Seen, Finally Heard: The Relational Impact of Self‐Discovery” identified that some participants found having a neurodivergent romantic partner was a supportive factor in feeling understood and potentially reducing the negative impact of ADHD traits on the relationship. This may reflect the theory of Double Empathy, which proposes that there is a mutual difficulty in understanding and communicating across neurotypes (Milton 2012). While the Double Empathy Problem originated and is mostly researched in the field of autism, it has the potential to explain social differences and difficulties in other neurotypes, such as ADHD or mental health difficulties (Livingston et al. 2025). Cross‐neurotype communication challenges are likely heightened in adults with ADHD who are also autistic. It would be valuable to explore the potentially supportive role of being in an intimate relationship with a fellow neurodivergent person for adults with ADHD.

### 4.1 Clinical Implications, Limitations, and Future Research

The findings of the present study offer several implications for clinical practice. First, the narrative accounts powerfully underscore the profound need for early detection of and intervention for ADHD. It does this both by highlighting the pervasive impact of its absence and the transformative effects of its presence. While the recognition of ADHD traits in adulthood often provides immense relief for individuals, it can also be accompanied by grief for missed supports, self‐recognition, and self‐compassion. To avoid this, it is imperative that clinical practitioners are appropriately equipped to recognize and support ADHD across the lifespan and across gender presentations.

Second, the need for self‐compassion, retrospective, present, and future is immense among people with ADHD. As the present study has shown, their eroded self‐concept and perceived low self‐efficacy characterize the way in which they navigate life and, indeed, relationships. Incorporating self‐compassion frameworks could replenish this psychological resource, which is commonly depleted in individuals with ADHD (Beaton et al. 2022b), such as Acceptance and Commitment Therapy, which has demonstrated preliminary effectiveness in improving self‐compassion in adults with ADHD (Seery et al. 2023, 2024). This may improve their relational experiences and their psychological well‐being, thus enhancing their overall quality of life.

This study also highlights the potential benefit of ADHD‐specific romantic relationships' interventions. As informed by the present study, interventions for ADHD‐involved romantic partnerships should go beyond the provision of psychoeducation and coping strategies and should provide a therapeutic space to explore how relational behaviors are interpreted and internalized by both partners. Several studies have explored the association between ADHD and attachment styles. A fearful avoidant attachment style has been shown to increase the likelihood of coping and responding with negative emotions via negative urgency (Faulkner 2024). Similarly, an anxious attachment style has been shown to mediate the relationship between ADHD traits and avoidance‐oriented coping (Al‐Yagon et al. 2020) and moderate the relationship between ADHD and depression and anxiety symptoms (Kordahji et al. 2021). Interventions for romantic dyads could incorporate addressing anxious attachment in adults with ADHD as a therapeutic goal (e.g., Paprocki and Baucom 2017). This could mitigate the risks of relational misunderstandings, conflict, and overall instability. It would be beneficial to conduct romantic relationship therapy with a neuroaffirmative framework (Chapman and Botha 2023).

This study presents various limitations. First, the reliance on self‐reported, retrospective accounts may introduce recall bias as well as a subjective interpretation of the relational challenges they recount.

Participants self‐reported their ADHD and indicated if they were self‐identified or had been formally diagnosed with ADHD. We did not verify ADHD status to confirm the presence of clinically significant traits. For example, Beaton et al. (Beaton et al. 2022a) did so in their qualitative study by employing the ADHD Self‐Report Scale (Kessler et al. 2005). The lack of confirmation of ADHD through clinical assessment or validation of diagnostic documents leads to several concerns and limitations. First, it is possible that some participants do not have ADHD, or their ADHD traits might be better explained by mental health difficulties or life circumstances. We could also not verify if participants had co‐occurring mental health difficulties, which represents challenges to the research, explored further in the limitations. Additionally, confirmation of ADHD through clinical assessments or validation of diagnostic documents would have supported us in identifying and comparing participants diagnosed in childhood and adolescence to those diagnosed as adults. Relatedly, ADHD is diagnosed at a higher rate in boys than girls during childhood, for several possible reasons including diagnostic practices and sociocultural expectations (Martin 2024; Martin et al. 2024). Therefore, gender and time of diagnosis are likely to affect themes related to intimate relationships in ADHD. Given the consequences, including in relationships, associated with a late identification of ADHD (Long and Coats 2022), this is an important consideration for future research exploring intimate relationships for adults with ADHD.

We did not invite participants to disclose any co‐occurring neurodivergent conditions, such as autism, or mental health difficulties (such as depression, anxiety, borderline personality disorder, or complex posttraumatic stress disorder) that may have impacted how they experience navigating romantic relationships. This represents a significant challenge of the research, as co‐occurring conditions may have affected experiences of relationships more so than ADHD, and themes may instead reflect the challenges of having ADHD and a co‐occurring condition in intimate relationships. Many adults with ADHD experience mental health difficulties (Choi et al. 2022; Hartman et al. 2023; Piñeiro‐Dieguez et al. 2016; Quenneville et al. 2022). As co‐occurring neurodivergence and mental health difficulties can impact experiences of relationships (e.g., Doyle et al. 2021; Howard et al. 2022; Khaw and Vernon 2025; Whisman et al. 2021), it would be valuable to explore qualitatively and quantitatively their impact on romantic relationships for adults with ADHD.

A significant limitation of the study is the wording of the open‐ended research question, which prompted participants to reflect on how ADHD “negatively” impacted their relationships. Other studies in the area of romantic relationships and ADHD have similarly focused on difficulties within relationships, such as Ben‐Naim et al. (2017), Merscher et al. (2025), and Steele et al. (2022). It would be valuable to also adopt a strengths‐based approach and consider protective factors that can be nurtured through interventions to support romantic relationships with an ADHD partner. The current study captures participants' experiences at a single point in time. Longitudinal research tracking the evolution of romantic relationships for individuals with ADHD over time could offer deeper insights into how ADHD traits impact their relational experiences and how these influence romantic patterns.

Additionally, relationship status and further details related to experiences of romantic relationships (e.g., demographic details regarding the prevalence of divorce, estimated number of romantic relationships) were not considered in the analysis. These factors may have informed participants' narratives in indirect and intersecting ways, which would have offered a meaningful contribution to our understanding of the relational experiences of individuals with ADHD. Likewise, we did not ask participants about their racial and ethnic identities and could not consider and explore the diversity of the sample.

We did not conduct gender‐specific exploration of findings to provide an overview of experiences of romantic relationships. However, research has suggested gendered differences, as women with ADHD report more self‐confidence issues, which might explain the descriptions of impacted self‐concept in the present study, and men report more relationship and family‐related impairments (Platania et al. 2025). Additionally, as boys are more likely to be diagnosed in childhood than girls, and girls are more likely to be diagnosed with mental health difficulties (Martin et al. 2024), late‐diagnosis may affect how men, women, and nonbinary people perceive their challenges in relation to ADHD and their romantic relationships. Therefore, further research is needed to explore qualitative gender‐specific experiences of ADHD in relationships as well as the gender‐specific experiences of their partners.

Similarly, the research on the impact of ADHD on romantic relationships has predominantly focused on participants in heterosexual relationships (Wymbs et al. 2021). The present study did not ask participants to specify if they were in a heterosexual or LGBTQ+ relationship or what their sexuality was. However, based on responses, participants were reporting on heterosexual partnerships. Given the overlap between neurodivergence and gender and sexual minority groups (Gayatri and Singh 2024; Goetz and Adams 2024) and that LGBTQ+ neurodivergent individuals have higher levels of mental health difficulties than neurotypical LGBTQ+ peers (Napoli 2025), it would be valuable for future research to qualitative explore how individuals who are LGBTQ+, and have ADHD experience romantic relationships, particularly in relation to gendered experiences.

Moreover, while the current study provides valuable insights into the lived experiences of adults with ADHD in romantic relationships, future empirical research would benefit from incorporating the perspectives of their romantic partners, involving both parties in the relationship to participate in the research. This would allow for a broader view of how ADHD traits are perceived, received, and navigated by both partners. This avenue of exploration could uncover naturally occurring coping strategies within relationships. Additionally, we did not ask participants what neurotype their partners were. It is clear this is an important factor, as it developed as a protective feature in relationships in the final theme. Future research should investigate partners' neurotypes and seek to explore the impact mixed, same, or similar neurotypes have on relationship satisfaction or distress for individuals with ADHD.

The current study consists of an Irish population, which contributes to a gap in the literature on the experiences of individuals with ADHD within an Irish context. However, this represents a challenge to the current body of research on intimate relationships in ADHD, as the majority of extant research has focused on Western populations. Future research should examine the topic through a multicultural lens that could offer insight into how different societal expectations and cultural influences shape the experiences of those with ADHD in romantic relationships.

## 5 Conclusion

This study offers a nuanced examination of how ADHD influences romantic relationships. It highlights the complex interplay between emotional regulation difficulties, executive functioning differences, self‐awareness, and relational dynamics. Through RTA, the study explored how individuals with ADHD interpret, navigate, and internalize these experiences. Three themes, “Too Much and Never Enough: The Emotional Rollercoaster of Rejection Sensitivity,” “The Struggle for Stability: ADHD's Battle Between Passion and Distraction,” and “Between Partner and Caregiver: The Emotional and Practical Strain in ADHD Relationships,” reflected the perceived impact of ADHD traits on romantic relationships, while “From Chaos to Clarity: The Role of Self‐Understanding in Love” emphasized the value of understanding experiences through the lens of ADHD and how that benefitted participants' views of themselves. While the study aligns with existing literature, it also showcases the individuality of ADHD experiences in romantic contexts. These findings reinforce the need for greater awareness, clinical support, and relationship‐focused interventions that acknowledge the unique relational needs of adults with ADHD. By addressing these challenges and leveraging the strengths of those with ADHD, future research and therapeutic frameworks can contribute to more fulfilling and sustainable romantic relationships for this population.
